# Plasma miR-122 and miR-3149 Potentially Novel Biomarkers for Acute Coronary Syndrome

**DOI:** 10.1371/journal.pone.0125430

**Published:** 2015-05-01

**Authors:** Xiangdong Li, Yuejin Yang, Laiyuan Wang, Shubin Qiao, Xiangfeng Lu, Yongjian Wu, Bo Xu, Hongfan Li, Dongfeng Gu

**Affiliations:** 1 Department of Epidemiology, State Key Laboratory of Cardiovascular Disease, Fuwai Hospital, National Center for Cardiovascular Diseases, Chinese Academy of Medical Sciences and Peking Union Medical College, Beijing, 100037, China; 2 Department of Cardiology, Fuwai Hospital, National Center for Cardiovascular Diseases, Chinese Academy of Medical Sciences and Peking Union Medical College, Beijing, 100037, China; Scuola Superiore Sant'Anna, ITALY

## Abstract

**Objective:**

We evaluated the potentiality of plasma microRNAs (miRNAs, or miRs) that were considered as novel biomarkers for acute coronary syndrome (ACS), including acute myocardial infarction (AMI) and unstable angina (UA).

**Methods and Results:**

We initially identified plasma miR-122, -140-3p, -144, -720, -1225-3p, -2861, and -3149 as candidate miRNAs associated with AMI (≥2 fold and P < 0.05) by comparing expression differences of miRNAs among AMI, non-coronary heart disease (non-CHD) and stable angina (SA) groups, using miRNA microarrays (n = 8 independent arrays in each group). Those seven plasma miRNAs were further examined with qRT-PCR analyses in two replications including 111 and 428 patients separately, and the results demonstrated that plasma miR-122, -140-3p, -720, -2861, and -3149 were elevated in the ACS group vs. the non-ACS (non-CHD + SA) group (P < 0.01). The area under the receiver operating characteristic curve (AUC) of the five miRNAs for ACS classification was 0.838, 0.818, 0.865, 0.852, and 0.670, respectively (all P < 0.001), while the values reached 0.843 and 0.925 when simultaneously with miR-122 and -3149 or with miR-122, -2861, and -3149 together (all P < 0.001). In plasma of pigs after coronary ligation, miR-122 was increased from 180 min to 240 min and miR-3149 was augmented from 30 min to 240 min compared with the sham pigs (all P < 0.05).

**Conclusion:**

Plasma miR-122, -140-3p, -720, -2861, and -3149 were associated with and potentially novel biomarkers for ACS.

## Introduction

Acute coronary syndrome (ACS), including acute myocardial infarction (AMI) and unstable angina (UA), is one of the most serious cardiovascular diseases and a major cause of mortality and morbidity worldwide. Timely revascularization therapy within 3 h after the onset of symptoms is recommended by guidelines to rescue the ischemic myocardium and, thereafter, could eventually reduce the mortality [1]. Therefore, an early and accurate discrimination of ACS patients is the guarantee for timely reperfusion therapy. Current diagnosis of ACS is based on typical symptoms, electrocardiogram (ECG), and cardiac troponin (cTn) I and T [1]. However, atypical symptoms are often occurred in elderly, non-ST-elevation MI (NSTEMI), and diabetes patients, and the ECG changes may be confusing as the presence of a preexisting left bundle branch block, pacemaker, and chronic ischemic cardiomyopathy or previous MIs [1, 2]. Furthermore, cardiac troponins can not be detected until 6 to 12 h after coronary occlusion, even high-sensitive troponins can only be detected until 3 h after myocardial infarction [1, 3]. Therefore, novel potential biomarkers are needed to improve ACS determination, specifically in patients with atypical symptoms and inconclusive ECG.

Recently, microRNAs (miRNAs, or miRs) have been demonstrated to be involved in the mechanism of ACS, including rupture of the atherosclerotic plaque [4, 5], platelet activation and aggregation [6, 7], and myocardial cells death after coronary occlusion [8]. Many miRNAs are expressed in a tissue-specific manner and can enter into plasma and urine [9], and evidences have showed the importance of circulating miRNAs as stable biomarkers for cancers [10, 11], raising the potential of miRNAs as ACS biomarker. Marked changes of miR-1 [12], -133a, -208, -208a [13], -208b, -499, and -499-5p [12] have been reported in animal models of AMI as well as in patients with ST-elevation MI (STEMI), and are considered as potential biomarkers in myocardial injury. However, most of these miRNAs can not be detected within 3h of AMI symptoms [13], and their diagnostic values are controversial in different studies [14]. Therefore, it is necessary and rational to search for novel miRNAs that changed earlier after AMI or ACS.

## Methods

For detailed methods, see S1 Appendix.

### Patients’ characteristics and blood sample collection

Blood samples were obtained in the cardiac catheterization laboratory from 16 healthy individuals (Health), 72 high-risk individuals (Highrisk), 81 stable angina (SA), 287 UA, and 115 AMI (including STEMI and NSTEMI) patients.

The protocol of this study was approved by the Ethics Committee of Fuwai Hospital, National Center for Cardiovascular Diseases, Chinese Academy of Medical Sciences and Peking Union Medical College, China. Written informed consent was obtained from each patient before enrolment.

### Animal experimental protocols

Thirty six Bama male minipigs (10 months old) were fed a high-cholesterol diet for 20 weeks. Then pigs were randomly assigned to Sham (n = 6) and AMI (n = 30) groups, and the AMI group was further divided into AMI with ventricular fibrillation (VF, AMI-VF) and AMI without VF (AMI-NVF) groups after the end of the operation. All pigs were anesthetized with a mixture of ketamine hydrochloride 700 mg and diazepam 30 mg intramuscularly and were continuously infused with the mixture (2 mg/kg per hour) intravenously. At the end of the experiment, the deeply anesthetized pig was killed by an injection of 15% KCl (1 ml/kg). Blood samples were collected before the operation and after 30, 60, 90, 120, 180, and 240 min of coronary ligation.

All animals received humane care in compliance with the Guide for the Care and Use of Laboratory Animals published by the National Institutes of Health, USA. The animal experimental protocols and procedures were approved by the Care of Experimental Animals Committee of Fuwai Hospital, National Center for Cardiovascular Diseases, Chinese Academy of Medical Sciences and Peking Union Medical College, China.

### Plasma collection and storage

Peripheral blood samples (5 mL) were collected in the cardiac catheterization laboratory, and were separated into plasma and cellular fractions within 2 h of collection.

### RNA preparation

Total RNA was isolated from plasma with TRIzol LS Reagent (Invitrogen, USA) according to the manufacturer’s protocol.

### miRNA profiling and validation

miRNAs profile in human plasma of AMI, Health, Highrisk and SA groups was determined by the human miRNA array (Agilent miRNAs microarray Version 16.0). The microarray dataset is publicly available at GEO database (GSE66752, http://www.ncbi.nlm.nih.gov/geo/query/acc.cgi?acc=GSE66752). The candidate miRNAs that differentially expressed in AMI patients were further validated by miRNA stem loop quantitative real-time polymerase chain reaction (qRT-PCR) technology.

### Plasma cTnI determination

Plasma cTnI concentrations were measured by ELISA assay according to the manufacturer’s protocol (Beckman Coulter, USA).

### Statistical analysis

Patients’ baseline characteristics of different groups were compared by One-way ANOVA and Fisher’s exact test. For microarray analysis, the Mann-Whitney unpaired test was used for the three pairwise comparisons (AMI vs. Health, AMI vs. Highrisk, and AMI vs. SA). For the data obtained by qRT-PCR, a widely used method to present relative expression of miRNA is the 2^-ΔΔct^ method. All values of miRNAs are expressed as mean ± SE. Comparisons of parameters among ≥ 3 groups were analyzed by oneway ANOVA, followed by post hoc testing with Bonferroni correction. Independent-sample T test was used for 2 group comparisons. All P values were two-sided and differences were considered statistically significant at a value of P < 0.05. The receiver operating characteristic (ROC) curves were established for classification of ACS and non-ACS patients. All statistical calculations were performed by the SPSS 20.0.

## Results

### miRNA array analysis

Expression profiles of 1205 human miRNAs were initially screened by microarray between the Health, Highrisk, SA, and AMI patients (n = 8 independent arrays in each group). No significant differences of clinical characteristics were found among the four groups (S1 Table).

Quantification revealed that plasma miR-122, -140-3p, -144, -720, -1225-3p, -2861, and -3149 were up-regulated ≥2 fold in the AMI patients compared with the Health, Highrisk, and SA patients (all P < 0.05). These 7 miRNAs were selected as candidate miRNAs for further testing via qRT-PCR in human samples (S2 Table). Because no differences of miRNAs expression were detected between the Health and Highrisk groups, the two groups were merged into one non-coronary heart disease (non-CHD) group in the subsequent study.

### Expression profiles of seven selected miRNAs in two replications

**Fig 1:**
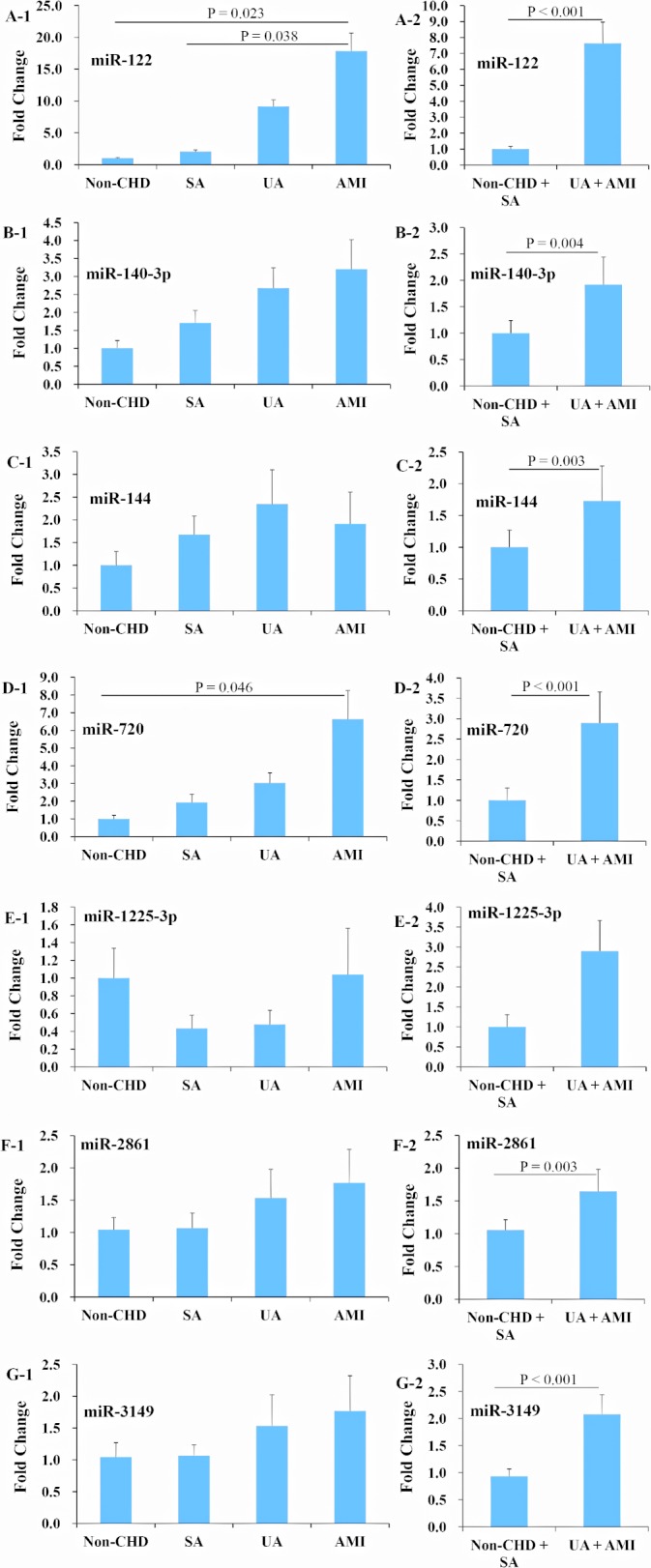
Expression profiles of candidate miRNAs in non-CHD, SA, UA, and AMI patients in a cohort of human samples. The 7 candidate miRNAs were first evaluated with qRT-PCR using 111 samples including 21 non-CHD, 30 SA, 30 UA, and 30 AMI patients. miR-122 and -720 were significantly up-regulated in the AMI group compared with the non-CHD group (A and D), and miR-122, -140-3p, -144, -720, -2861, and -3149 were increased in the acute coronary syndrome (ACS, UA + AMI) group compared with the non-ACS (non-CHD + SA) group (A, B, C, D, F, and G). Abbreviations: CHD = coronary heart disease, SA = stable angina, UA = unstable angina, AMI = acute myocardial infarction.

The seven candidate miRNAs were first tested with qRT-PCR in 111 human plasma samples, including 21 non-CHD, 30 SA, 30 UA, and 30 AMI patients, and no significant differences of clinical characteristics were found among the four groups (S3 Table). The results showed that miR-122 and -720 were significantly increased by 17.8 and 6.6 fold in the AMI group compared with the non-CHD group, respectively (all P < 0.05) (Fig 1, Panels A and D). The ACS (UA + AMI) group had higher levels of miR-122, -140-3p, -144, -720, -2861, and -3149 than did the non-ACS (non-CHD + SA) group, based on the classification of coronary plaque stability (all P < 0.01) (Fig 1, Panels A, B, C, D, F, and G). Because miR-144 detection rate was very low (only 64.0%), and no significant differences of the miR-1225-3p expression levels were detected between the ACS and non-ACS patients, these two miRNAs were excluded in the next stage of study.

**Fig 2:**
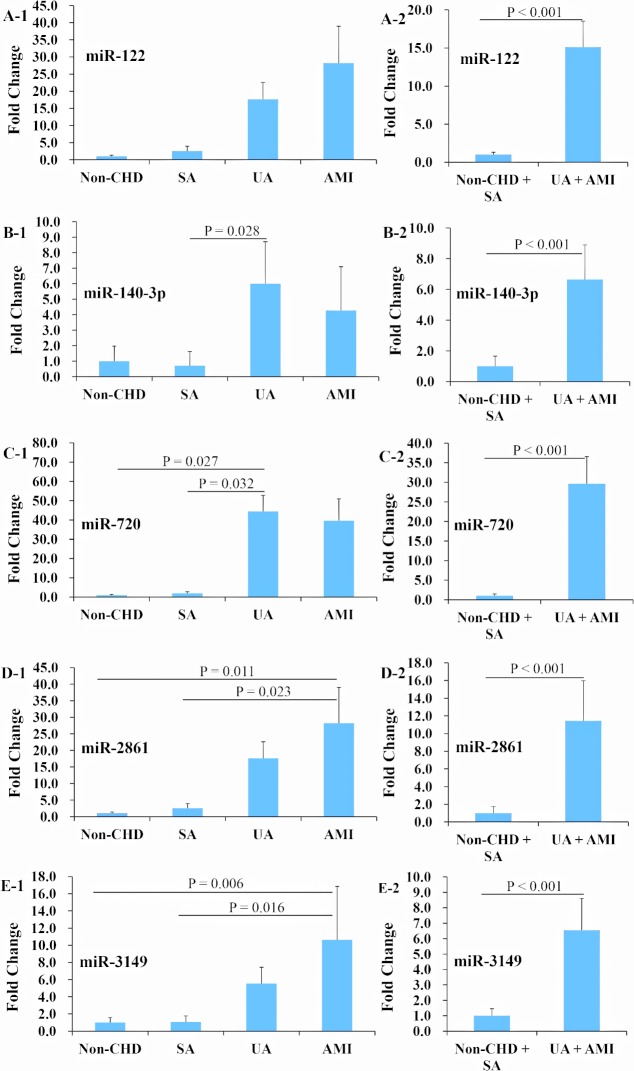
Expression profiles of miR-122, -140-3p, -720, -2861, and -3149 in non-CHD, SA, UA, and AMI patients in another cohort of human samples. The expression profiles of miR-122, -140-3p, -720, -2861, and -3149 were further tested in 428 additional plasma samples, including 51 non-CHD, 43 SA, 257 UA, and 77 AMI patients with qRT-PCR. The results showed that plasma miR-122, -140-3p, -720, -2861, and -3149 were significantly up-regulated in the acute coronary syndrome (ACS, UA + AMI) group versus the non-ACS (non-CHD + SA) group (A to E); and miR-2861 and -3149 were up-regulated in the AMI group versus the non-CHD and SA groups (D and E). Abbreviations as in Fig 1.

The expression profiles of miR-122, -140-3p, -720, -2861, and -3149 were further examined with qRT-PCR in 428 additional subjects’ plasma samples, including 51 non-CHD, 43 SA, 257 UA, and 77 AMI patients, and no significant differences of clinical characteristics were found among the four groups (S4 Table). The results revealed that the expression levels of miR-122, -140-3p, -720, -2861, and -3149 were higher in the ACS group compared with the non-ACS group (all P < 0.001), and particularly miR-2861 and -3149 were dramatically elevated in the AMI group versus the non-CHD and SA groups (all P < 0.05) (Fig 2).

### miRNAs expression profiles for ACS versus non-ACS in the training data set

**Fig 3:**
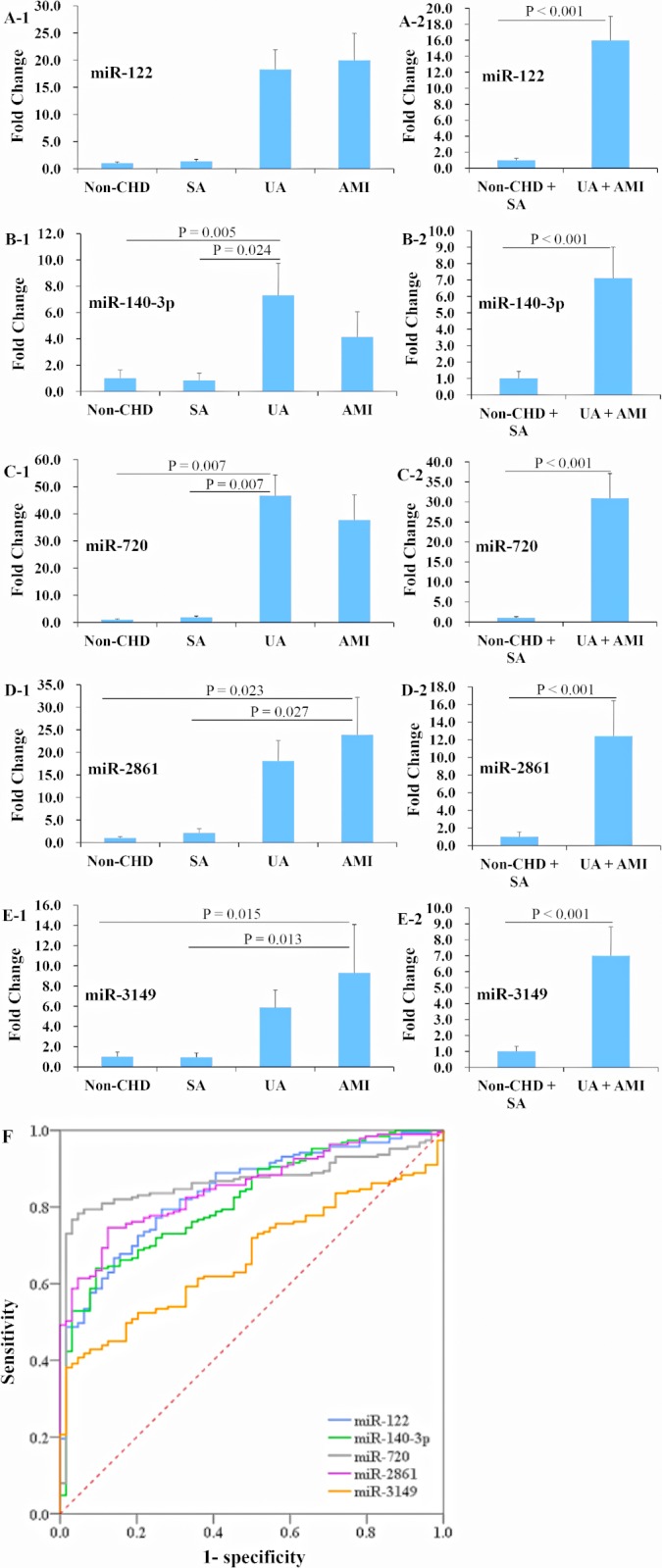
Expression profiles of plasma miR-122, -140-3p, -720, -2861, and -3149 in ACS and non-ACS patients in the training data set. A total of 539 plasma samples were used for the training data set in deriving the miRNA panel for use in the classification of ACS and non-ACS. The expression levels of miR-122, -140-3p, -720, -2861, and -3149 were dramatically elevated in the ACS (UA + AMI) group compared with the non-ACS (non-CHD + SA) group (A to E). The determination values of these five plasma miRNAs were analyzed by ROC curves (F). Abbreviations as in Fig 1.

A total of 539 human plasma samples from the two replications, including 394 ACS patients and 145 non-ACS patients, were used as the resource of training data set for the construction of the miRNA panel for the classification of ACS and non-ACS patients. The expression of miR-122, -140-3p, -720, -2861, and -3149 was increased respectively by 20.7, 14.7, 49.3, 27.6, and 12.5 fold in the ACS group compared with the non-ACS group (all P < 0.001) (Fig 3 and Table 1). The accuracy of these five miRNAs for ACS determination was evaluated by the ROC analysis, and the area under the ROC curve (AUC) for these miRNAs was 0.838 (95%CI 0.787–0.89), 0.818 (95%CI 0.762–0.873), 0.865 (95%CI 0.818–0.913), 0.852 (95%CI 0.806–0.899), and 0.670 (95%CI 0.604–0.736), respectively (all P < 0.001) (Table 1). The AUC reached 0.843 (95%CI 0.791–0.894) and 0.925 (95%CI 0.9–0.95) respectively for ACS determination when simultaneously with miR-122 and -3149 or with miR-122, -2861, and -3149 together (all P < 0.001).

**Table 1:** miRNA expression profile and classification performance for ACS versus non-ACS in the training data set including 394 ACS and 145 non-ACS patients.

miRNAs	Fold Change	AUC	95%CI	P values
miR-122	20.7	0.838	0.787–0.89	< 0.001
miR-140-3p	14.7	0.818	0.762–0.873	< 0.001
miR-720	49.3	0.865	0.818–0.913	< 0.001
miR-2861	27.6	0.852	0.806–0.899	< 0.001
miR-3149	12.5	0.670	0.604–0.736	< 0.001
miR-122 + -3149	-	0.843	0.791–0.894	< 0.001
miR-122 + -2861+ -3149	-	0.925	0.9–0.95	< 0.001

Abbreviations: AUC = area under the receiver operating characteristic curve; CI = confidence interval.

### miRNAs plasma levels in AMI minipigs

**Fig 4:**
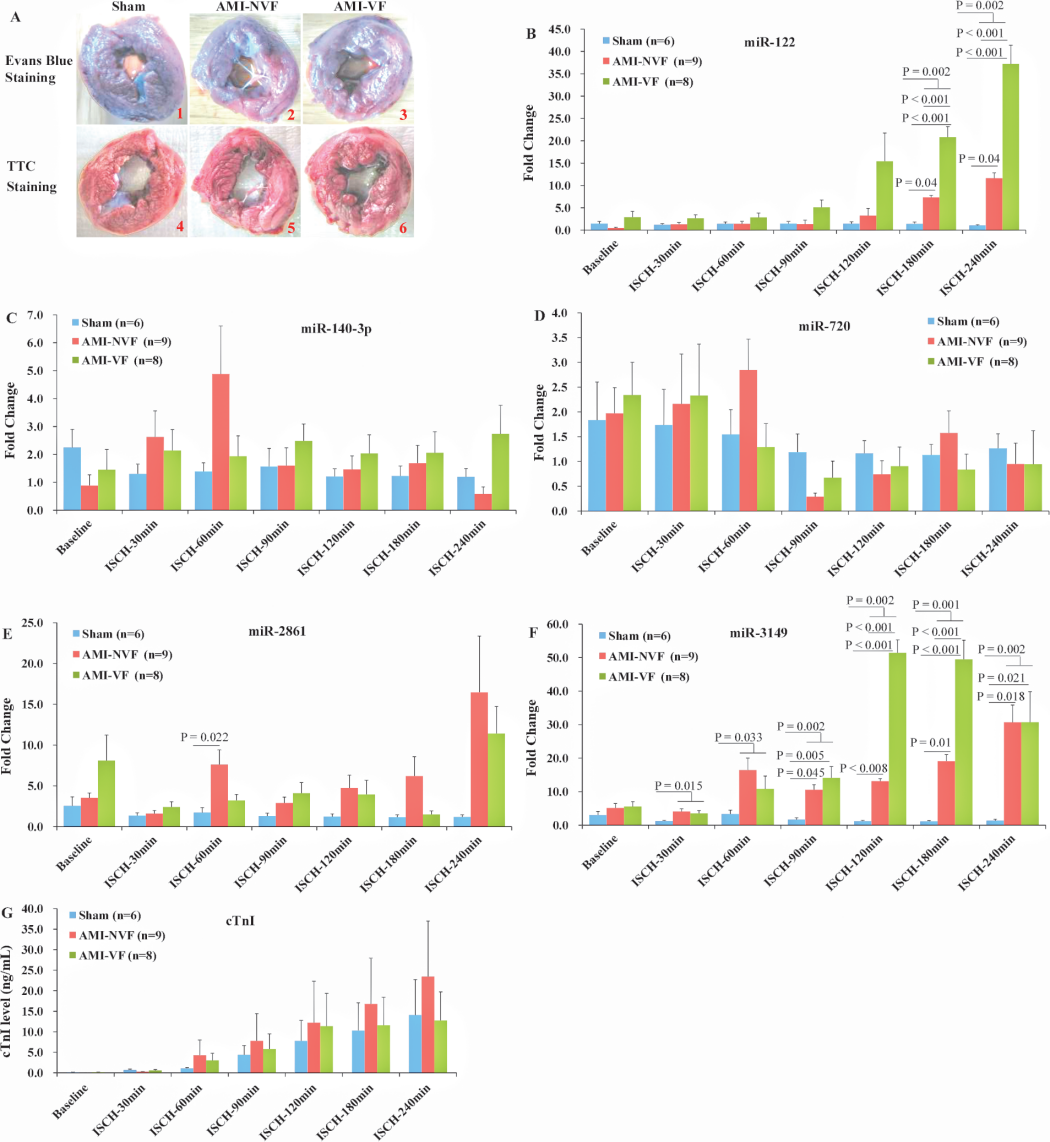
Expression profiles of circulating miR-122, -140-3p, -720, -2861, and -3149 in swine AMI model. The myocardium unstained by Evans blue dye represented ischemic myocardium, and the triphenyltetrazolium chloride (TTC)-unstained white myocardium was identified as infarct zone, indicating the success of AMI model (A). Compared with the sham group, miR-122 plasma levels were up-regulated after 180 and 240 min of coronary occlusion (B), and miR-3149 plasma levels were elevated from 30 min to 240 min after myocardial ischemia (F). No significant differences of plasma levels of cTnI were detected within 240 min of myocardial infarction versus the sham group (G). Abbreviations: AMI-NVF = AMI without ventricular fibrillation, AMI-VF = AMI with ventricular fibrillation, ISCH = ischemia.

Animal experiments were conducted to assess whether the effects of ACS on miRNAs plasma levels in humans were observed in a swine model of AMI. Evans blue and triphenyltetrazolium chloride (TTC) staining indicated the success of AMI model (Fig 4, Panel A). The results demonstrated that, after 180 and 240 min of coronary occlusion, plasma miR-122 levels were rapidly increased compared with the sham group (P = 0.002), and were higher as well in the AMI-VF group than in the AMI-NVF group (P < 0.001) (Fig 4, Panel B). Furthermore, plasma miR-3149 levels were sharply augmented from 30 min to 240 min after myocardial ischemia compared to the sham group (all P < 0.05), and were higher as well in the AMI-VF group than in the AMI-NVF group after 120 and 180 min of coronary ligation (all P < 0.001) (Fig 4 Panel F). However, no significant differences were detected in plasma levels of miR-140-3p, -720, -2861, and cTnI within 240 min after coronary occlusion compared with the sham group (all P > 0.05) (Fig 4, Panels C, D, E, and G).

## Discussion

Ischemic heart disease accounts for approximately 30% of all deaths, causing 1.4 million deaths in the developed world and 5.7 million deaths in developing regions [15]. While about 20% of these deaths die from ACS, leading ACS is the first cause of human mortality and morbidity in the world [16], underscoring the need for novel biomarkers for its determination and therapies.

In this study, we reported that the levels of circulating miR-122, -140-3p, -720, -2861, and -3149 were significantly up-regulated in human ACS, in comparison with the non-ACS patients. The ability of the five miRNAs to distinguish the ACS patients from the non-ACS patients was shown by the ROC curves with the AUC of 0.838, 0.818, 0.865, 0.852, and 0.670, respectively (Table 1), and the AUC even reached 0.925 when determining ACS simultaneously with miR-122, -2861, and -3149. In the present study, age, gender and clinical features such as hypertension, diabetes, smoking, alcohol consumption, and CHD family history were comparable among different groups, so effects of possible bias from patient selection on our miRNAs findings were limited (S1, S3, and S4 Tables). Our clinical patients’ findings thus, implied that miR-122, -140-3p, -720, -2861, and -3149 are associated with ACS. On the other hand, animal study with swine AMI model showed that, after coronary ligation, plasma miR-122 levels were up-regulated from 180 min to 240 min, and circulating miR-3149 levels were also up-regulated from 30 min to 240 min. In contrast, the plasma cTnI levels were remained below the cut-off value detectable even after 240 min of AMI. Therefore, our results showed that high levels of miR-122 and -3149 may indicate AMI.

miR-122, regarded as liver-specific [17], plays crucial roles in lipid metabolism [18], and was associated with the presence and severity of CHD in hyperlipidemia patients [19]. miR-122 was expressed at very low levels or undetectable in the heart [12], but was increased in the border and ischemic areas in mice with AMI [12], and was elevated in the plasma of patients with acute heart failure [20]. However, miR-122 levels were not increased in the plasma of AMI patients and mice models, and even inversely associated with the incidence of MI [12]. The inconsistency of the association between plasma miR-122 level and AMI in different studies may be partially explained by the different characteristics of patients and animals among those studies. In previous human studies, the AMI patients included only STEMI patients but not NSTEMI patients, and the miRNAs expression levels were only compared between the patients with AMI or ACS and the healthy individuals or non-CHD patients, but not compared among different groups of the high risk subjects, SA, and UA patients. In previous animal studies, the AMI model was made on common diet-fed small animals, and the animals were relatively young (< 4 month); while in our animal study, the AMI model was made on high-cholesterol diet-fed large animals, and the animals were relatively old (10 month). Therefore, the animal model in our study could mimic the characteristics of CHD patients better than that in the previous studies.

miR-140-3p has been reported to be increased in CHD [21]. miR-720 has been found to be involved in tumor formation, invasion, and migration [22]. miR-2861 was up-regulated in basal cell [23] and papillary thyroid carcinoma [24], and played a positive role in osteoblast differentiation and chordoma development [25]. miR-3149 was down-regulated in gastric cancer [26]. However, the expression profiles of these miRNAs have not been reported yet in ACS patients and AMI animals. In our study we identified that these five miRNAs were drastically up-regulated in ACS patients compared with non-ACS patients, and the plasma miR-3149 was expeditiously increased as early as 30 min in pigs after coronary ligation, indicating its association with early ACS.

In addition, our animal study found that the plasma levels of miR-122 and -3149 were not only higher in the AMI pigs in comparison with the sham pigs, but also higher in the AMI-VF pigs versus the AMI-NVF pigs. The upregulated miR-21 has been reported to promote atrial fibrillation (AF) in MI-induced heart failure in rats [27]. miR-29 was decreased in dogs with ventricular tachypacing-induced congestive heart failure (CHF) and in patients with CHF or AF [28]. miR-328 was elevated in AF dogs and patients [29]. These reports suggested that miRNAs were involved in the mechanism of AF, and may be potential AF markers or promising drug targets during heart failure after myocardial infarction. Our study further suggested that miRNAs may also play a role in the development of VF, and elevated miR-122 and -3149 may be associated with VF after myocardial infarction as well.

It should be noted that the levels of cTnI were not significantly different within 240 min after coronary ligation between the sham and AMI pigs with the success of AMI model documented clearly by the triphenyltetrazolium chloride (TTC) staining. Previous studies have demonstrated that the cTnI levels in blood of AMI patients began to rise after 4–8 h of myocardial injury [30]. But in other studies of small animals, cTnI was higher than that of the sham animals within 1 h in mice [13], even 15 min in rats [12] after coronary ligation. These findings implied that AMI models made on common diet-fed small animals were hard to mimic the characteristics of AMI patients, while the animal model in our study could better mimic the characteristics of AMI patients. Another explanation is that the cTnI elevation caused by skeletal muscles injury during operation diminished the cTnI elevation caused by myocardial injury, because cTnI was expressed in skeletal muscles as well [3].

The potential limitations should be addressed. First, the sample size of current study is relatively small. Second, the generalizability of our findings is limited by the demographic differences and treatment characteristics of ACS patients. In addition, it would be ideal to have information on the time course for corresponding miRNA quantification in AMI patients. Therefore, further studies with larger sample size and patients with various CHD subtypes are needed to validate the diagnostic value of plasma miR-122 and -3149 for ACS.

In conclusion, plasma miR-122 and miR-3149 are associated with ACS and might be potentially novel biomarkers for accelerating the diagnosis of ACS patients in emergency department.

## Supporting Information

S1 Appendix Supporting Materials and Methods. (DOCX)

S1 Table Clinical characteristics of different patient groups with microarray analysis. (DOC)

S2 Table Differentially expressed miRNAs in AMI versus Health, Highrisk, and SA patients by microarray analysis. (DOC)

S3 Table Clinical characteristics of different patient groups in the first cohort with expression profiles of seven selected miRNAs by qRT-PCR. (DOC)

S4 Table Clinical characteristics of different patient groups in the second cohort with expression profiles of five selected miRNAs by qRT-PCR. (DOC)
