# Supplementary material for: Plasma miR-122 and miR-3149 Potentially Novel Biomarkers for Acute Coronary Syndrome
Source: PLoS One. 2015 May 1;10(5):e0125430. doi: 10.1371/journal.pone.0125430 (PMC4416808; doi:10.1371/journal.pone.0125430)
Supplement: S1 Appendix — (DOCX) [file pone.0125430.s001.docx]

### S1 Appendix. Supporting Materials and Methods

### Materials and Methods

### Population

We assessed 115 acute myocardial infarction (AMI) and 456 non-AMI patients with chest distress and pain admitted to Department of Cardiology in FUWAI Hospital, National Center for Cardiovascular Diseases, Beijing, China, between February 2012 and August 2012. AMI patients, including ST-elevation MI (STEMI) and non-ST-elevation MI (NSTEMI) patients, were clinically diagnosed by acute ischemic-type chest pain, electrocardiogram (ECG) change, biochemical markers (cTnI > 0.1 ng/mL), and coronary angiography according to newly developed universal definition of MI [[1](#_ENREF_1)]. The non-AMI patients with distress and chest pain were separated by the outcomes of coronary angiography, including 368 patients with coronary heart disease (CHD) and 88 patients without CHD (non-CHD). Among the 368 CHD patients, 81 and 287 patients were identified as stable angina (SA) and unstable angina (UA) based on typical chest pain [[2](#_ENREF_2)], respectively. SA was defined as ischemic symptoms onset only with physical activities, without dynamic ECG changes and cTnI < 0.03 ng/mL. UA was defined as ischemic symptoms which can be frequent and onset with little or no physical exertion, with or without dynamic ECG changes, and cTnI < 0.03 ng/mL. Among the 88 non-CHD patients, 16 were considered to be healthy individuals (Health) that had ≤ 2 risk factors for CHD, and the other 72 patients were regarded as high-risk individuals (Highrisk) that had ≥ 3 risk factors for CHD. Patients were excluded if they had a known history of autoimmune disease, acute or chronic infection, severe hepatic or renal dysfunction, and evidence of malignant disease. To investigate the characters of these miRNAs as potential biomarkers of ACS, the receiver operating characteristic (ROC) analysis was performed on data from ACS and non-ACS patients.

The protocol of this study was approved by the Ethics Committee of FUWAI Hospital, National Center for Cardiovascular Diseases, Chinese Academy of Medical Sciences and Peking Union Medical College, China. Written informed consent was obtained from each patient before enrolment.

**Animal experimental protocols**

Thirty six Bama male minipigs (10 months old) weighing 25 to 35 kg were fed a high-cholesterol diet containing 3% cholesterol, 15% lard, 6.0％ arachisoil, 5％ plantation white sugar, 0.5％ bile salts, and 0.025 μg/kg/d of nicotine for 20 weeks, without limitation of activities. Then pigs were randomly assigned to 2 groups: (1) AMI (n = 30): all animals were underwent 4 h occlusion of left anterior descending coronary artery (LAD), and these animals were further divided into AMI with ventricular fibrillation (VF, AMI-VF) and AMI without VF (AMI-NVF) groups after the end of the operation; (2) Sham (n = 6): the LAD was only encircled by a suture, but not occluded. All pigs were anesthetized with a mixture of ketamine hydrochloride 700 mg and diazepam 30 mg intramuscularly and were continuously infused with the mixture (2 mg/kg per hour) intravenously. Each animal was ventilated with a high-frequency jet ventilator (SV900; Siemens-Elema, Solna, Sweden). Body temperature was monitored by a rectal thermometer and maintained constant between 37℃ and 38℃ by a heating pad. Internal electric defibrillator with 20 joule was used when ventricular fibrillation lasted for more than 10 seconds. At the end of the expeirment, the deeply anesthetized pig was killed by an injection of 15% KCl (1 ml/kg). Blood samples were collected before the operation and after 30, 60, 90, 120, 180, and 240 min of ischemia for the detection of miRNAs expression profiles.

All animals received humane care in compliance with the Guide for the Care and Use of Laboratory Animals published by the National Institutes of Health, USA. The animal experimental protocols and procedures were approved by the Care of Experimental Animals Committee of FUWAI Hospital, National Center for Cardiovascular Diseases, Chinese Academy of Medical Sciences and Peking Union Medical College, China.

**Plasma collection and storage**

To insure that all of participants were finally diagnosed according to coronary angiography, peripheral blood samples (5 mL) for miRNAs detection were collected into EDTA-containing tubes from the patients in the cardiac catheterization laboratory. All blood samples were kept at 4˚C and separated into plasma and cellular fractions within 2 h of collection by centrifugation at 1500 g for 15 min. Then the plasma was transferred to a fresh RNase/DNase-free Eppendorf tube and centrifuged at 2500 g at 4°C for 10 min to pellet the remaining cellular debris. The supernatant was transferred to RNase/DNase-free tubes and stored at −80°C. The samples with hemolysis were excluded.

**RNA preparation**

Total RNA was isolated from plasma with TRIzol LS Reagent (Invitrogen, USA) according to the manufacturer’s protocol. In brief, total RNA was purified and ultimately eluted into 20 μL of RNase-free water. RNA quantity was assessed by NanoDrop (NanoDrop Products, Wilmington, Del., USA).

**miRNA microarray**

miRNAs profile in human plasma was determined by the human miRNA array (Agilent miRNAs microarray Version 16.0) that includes 1205 human and 142 human viral miRNAs (Sanger miRBase, release 16.0). The microarray dataset is publicly available at GEO database (GSE66752, <http://www.ncbi.nlm.nih.gov/geo/query/acc.cgi?acc=GSE66752>). Each slide is formatted with 8 identical arrays and hybridized with 100 ng Cy3-labeled RNA according to the protocols in the Agilent miRNA microarray system. Slides were scanned by Agilent Microarray Scanner (Cat#G2565BA, Agilent technologies, USA) and Feature Extraction software 10.7 (Agilent technologies, USA) with default settings. Raw data were normalized by Quantile algorithm, Gene Spring Software 11.0 (Agilent technologies, USA). The raw signals obtained for single-color CY3 hybridization were normalized by a stable endogenous control miR-1228 [[3](#_ENREF_3)], which was stably expressed in all samples. After that, a log transform with base 2 was performed. A sample that showed intra-array coefficients of variation (CV) across replicated spots on an array above 15% or positive signals less than 5% was considered to be unreliable and excluded from further analysis. A detectable miRNA was defined as the miRNA had positive signals on microarrays in > 50% of plasma samples from any one of the four categories including AMI, Health, Highrisk and SA groups.

**Quantitative real-time polymerase chain reaction analysis**

### The expression of specific miRNAs was analyzed by miRNA stem loop quantitative real-time polymerase chain reaction (qRT-PCR) technology on an ABI PRISM7900 system according to the protocol of the manufacturer (Applied Biosystems, USA). TaqMan qRT-PCR was carried out with FastStart Universal Probe Master Mix (Roche, Germany) to detect the expression levels of miR-122, -140-3p, -144, -720, -1225-3p, and -1228 (Assay ID: 002245, 002234, 197375, 002895, 002766, and 002919, respectively, Applied Biosystems, USA). qRT-PCR was carried out with Fast SYBR Green Select Master Mix (Applied Biosystems, USA) to detect the expression levels of miR-2861 (RT primer CTCAACTGGTGTCGTGGAGTCGGCAATTCAGTTGAGCCGCCCAC, forward primer ACACTCCAGCTGGGGGGGCCTGGCGGT, and reverse primer TGGTGTCGTGGAGTCG, Nanjing BioSteed BioTechnologies Co., Ltd, China), and -3149 (RT primer CTCAACTGGTGTCGTGGAGTCGGCAATTCAGTTGAGATACACAC, forward primer ACACTCCAGCTGGGTTTGTATGGATATGTGT, and reverse primer TGGTGTCGTGGAGTCG, Nanjing BioSteed BioTechnologies Co., Ltd, China). cDNA was synthesized from 20 ng of total RNA using a cDNA synthesis kit (Roche, Germany) at 16°C for 30 min, 42°C for 30 min, and denaturation of the enzyme at 85°C for 5 min. The assay was run in 20 µL triplicate reactions for each case to allow for assessment of technical variability. Briefly, the reactions were incubated at 50°C for 2 min, 95°C for 10 min, followed by 45 cycles of 95°C for 15 s, 60°C for 60 s. The relative expression level for each miRNA was computed using the comparative CT method. The Ct values from qRT-PCR assays greater than 45 were treated as 45. Ct values were converted into copy numbers (copy no. = 2 (−Ct)) and normalized to miR-1228. The data obtained by qRT-PCR were translated into log2 (relative level), and the outliers were excluded using scatterplot during statistical analysis.

### References:

1. Hamm CW, Bassand JP, Agewall S, et al. Esc guidelines for the management of acute coronary syndromes in patients presenting without persistent st-segment elevation: The task force for the management of acute coronary syndromes (acs) in patients presenting without persistent st-segment elevation of the european society of cardiology (esc). Eur Heart J. 2011;32:2999-3054.

2. Stellos K, Gawaz M. Platelet interaction with progenitor cells: Potential implications for regenerative medicine. Thromb Haemost. 2007;98:922-929.

3. Zhou J, Yu L, Gao X, et al. Plasma microrna panel to diagnose hepatitis b virus-related hepatocellular carcinoma. J Clin Oncol. 2011;29:4781-4788.
