# Supplementary material for: Plasma miR-122 and miR-3149 Potentially Novel Biomarkers for Acute Coronary Syndrome
Source: PLoS One. 2015 May 1;10(5):e0125430. doi: 10.1371/journal.pone.0125430 (PMC4416808; doi:10.1371/journal.pone.0125430)
Supplement: S1 Table — (DOC) [file pone.0125430.s002.doc]

**Table S1. Clinical characteristics of different patient groups with microarray analysis.**

| **Variable** | **Health, n = 8** | **Highrisk, n = 8** | **SA, n = 8** | **AMI, n = 8** | **P values** |
| --- | --- | --- | --- | --- | --- |
| Gender, male/female | 4/4 | 6/2 | 5/3 | 5/3 | 0.793 |
| Age, yrs | 56.0 ± 3.9 | 58.1 ± 2.1 | 59.6 ± 3.6 | 63.9 ± 2.3 | 0.337 |
| Hypertension, n | 2 | 7 | 8 | 7 | 1.000 |
| Hyperlipidemia, n | 5 | 7 | 7 | 5 | 1.000 |
| Diabetes, n | 0 | 4 | 5 | 4 | 1.000 |
| Stroke, n | 0 | 0 | 0 | 0 | 1.000 |
| Smoking, n | 1 | 6 | 3 | 3 | 1.000 |
| Alcohol consumption, n | 0 | 6 | 2 | 3 | 1.000 |
| CHD family history, n | 2 | 1 | 2 | 1 | 1.000 |

Abbreviations: CHD = coronary heart disease, SA = stable angina, AMI = acute myocardial infarction.
