# Supplementary material for: Plasma miR-122 and miR-3149 Potentially Novel Biomarkers for Acute Coronary Syndrome
Source: PLoS One. 2015 May 1;10(5):e0125430. doi: 10.1371/journal.pone.0125430 (PMC4416808; doi:10.1371/journal.pone.0125430)
Supplement: S2 Table — (DOC) [file pone.0125430.s003.doc]

**Table S2. Differentially expressed miRNAs in AMI versus Health, Highrisk, and SA patients by microarray analysis.**

| **miRNAs** | **AMI vs. Health** | | **AMI vs. Highrisk** | | **AMI vs. SA** | |
| --- | --- | --- | --- | --- | --- | --- |
|  | **Fold Change** | **P values** | **Fold Change** | **P values** | **Fold Change** | **P values** |
| hsa-miR-106b | 2.1 | 0.672 | 2.4 | 0.248 | 2.1 | 0.626 |
| **hsa-miR-122** | **4.0** | **0.036** | **3.0** | **0.015** | **3.6** | **0.045** |
| **hsa-miR-1225-3p** | **2.3** | **0.038** | **2.6** | **0.011** | **2.2** | **0.039** |
| hsa-miR-126 | 1.8 | 0.034 | 1.3 | 0.055 | 2.1 | 0.799 |
| hsa-miR-130b | 2.6 | 0.529 | 1.7 | 0.688 | 3.0 | 0.551 |
| hsa-miR-133b | 3.5 | 0.753 | 16.8 | 0.138 | 16.4 | 0.148 |
| **hsa-miR-140-3p** | **4.0** | **0.021** | **4.7** | **0.004** | **4.9** | **0.012** |
| **hsa-miR-144** | **3.7** | **0.036** | **3.1** | **0.049** | **3.7** | **0.047** |
| hsa-miR-1471 | 5.2 | 0.348 | 7.0 | 0.538 | 7.0 | 0.538 |
| hsa-miR-15b | 1.8 | 0.674 | 2.1 | 0.443 | 2.1 | 0.558 |
| hsa-miR-1825 | 2.0 | 0.558 | 3.1 | 0.182 | 3.4 | 0.979 |
| hsa-miR-192 | 2.2 | 0.834 | 2.1 | 0.439 | 1.9 | 0.015 |
| hsa-miR-19a | 1.8 | 0.841 | 1.5 | 0.843 | 1.9 | 0.017 |
| hsa-miR-208b | 15.0 | 0.224 | 15.1 | 0.219 | 14.7 | 0.232 |
| hsa-miR-223 | 2.1 | 0.916 | 2.3 | 0.646 | 2.8 | 0.247 |
| hsa-miR-2276 | 2.2 | 0.834 | 2.3 | 0.177 | 2.2 | 0.240 |
| hsa-miR-23a | 3.6 | 0.674 | 2.2 | 0.492 | 2.3 | 0.156 |
| hsa-miR-24 | 2.3 | 0.600 | 2.4 | 0.233 | 1.6 | 0.197 |
| **hsa-miR-2861** | **4.8** | **0.040** | **3.0** | **0.029** | **2.1** | **0.027** |
| hsa-miR-29c | 2.5 | 0.169 | 2.1 | 0.490 | 2.1 | 0.987 |
| hsa-miR-30a | 2.8 | 0.753 | 2.1 | 0.153 | 6.0 | 0.183 |
| hsa-miR-30d | 1.8 | 0.035 | 2.5 | 0.150 | 1.6 | 0.196 |
| hsa-miR-3141 | 3.0 | 0.384 | 2.4 | 0.247 | 2.7 | 0.192 |
| **hsa-miR-3149** | **4.0** | **0.034** | **2.9** | **0.046** | **3.0** | **0.009** |
| hsa-miR-3188 | 2.4 | 0.834 | 2.4 | 0.143 | 2.3 | 0.186 |
| hsa-miR-3195 | 3.4 | 0.294 | 3.0 | 0.700 | 2.6 | 0.248 |
| hsa-miR-3610 | 2.2 | 0.102 | 1.9 | 0.047 | 1.6 | 0.162 |
| hsa-miR-3612 | 3.0 | 0.401 | 3.3 | 0.182 | 3.3 | 0.182 |
| hsa-miR-423-5p | 6.4 | 0.141 | 2.7 | 0.230 | 9.3 | 0.089 |
| hsa-miR-4313 | 2.4 | 0.674 | 2.6 | 0.429 | 3.4 | 0.726 |
| hsa-miR-484 | 2.1 | 0.834 | 2.1 | 0.418 | 2.1 | 0.220 |
| hsa-miR-574-5p | 6.3 | 0.208 | 3.8 | 0.330 | 2.4 | 0.333 |
| hsa-miR-584 | 2.9 | 0.674 | 2.6 | 0.350 | 1.8 | 0.211 |
| **hsa-miR-720** | **3.6** | **0.012** | **3.2** | **0.010** | **2.9** | **0.049** |
| hsa-miR-939 | 4.1 | 0.362 | 3.2 | 0.605 | 3.2 | 0.605 |

Abbreviations: SA = stable angina, AMI = acute myocardial infarction.
