# Supplementary material for: Plasma miR-122 and miR-3149 Potentially Novel Biomarkers for Acute Coronary Syndrome
Source: PLoS One. 2015 May 1;10(5):e0125430. doi: 10.1371/journal.pone.0125430 (PMC4416808; doi:10.1371/journal.pone.0125430)
Supplement: S3 Table — (DOC) [file pone.0125430.s004.doc]

#### Table S3. Clinical characteristics of different patient groups in the first cohort with expression profiles of seven selected miRNAs by qRT-PCR.

| **Variable** | **non-CHD, n = 21** | **SA, n = 30** | **UA, n = 30** | **AMI, n = 30** | **P values** |
| --- | --- | --- | --- | --- | --- |
| Gender, male/female | 12/9 | 16/14 | 25/5 | 24/6 | 1.000 |
| Age, yrs | 54.0 ± 1.9 | 59.2 ± 1.8 | 60.4 ± 1.9 | 60.0 ± 2.0 | 0.120 |
| Hypertension, n | 13 | 21 | 18 | 12 | 1.000 |
| Hyperlipidemia, n | 13 | 27 | 25 | 24 | 1.000 |
| Diabetes, n | 2 | 4 | 10 | 8 | 1.000 |
| Stroke, n | 2 | 2 | 7 | 2 | 1.000 |
| Smoking, n | 11 | 12 | 19 | 14 | 1.000 |
| Alcohol consumption, n | 9 | 7 | 12 | 9 | 1.000 |
| CHD family history, n | 2 | 7 | 4 | 5 | 1.000 |

Abbreviations: CHD = coronary heart disease, SA = stable angina, UA = unstable angina, AMI = acute myocardial infarction.
