# Supplementary material for: Plasma miR-122 and miR-3149 Potentially Novel Biomarkers for Acute Coronary Syndrome
Source: PLoS One. 2015 May 1;10(5):e0125430. doi: 10.1371/journal.pone.0125430 (PMC4416808; doi:10.1371/journal.pone.0125430)
Supplement: S4 Table — (DOC) [file pone.0125430.s005.doc]

#### Table S4. Clinical characteristics of different patient groups in the second cohort with expression profiles of five selected miRNAs by qRT-PCR.

| **Variable** | **non-CHD, n = 51** | **SA, n = 43** | **UA, n = 257** | **AMI, n = 77** | **P values** |
| --- | --- | --- | --- | --- | --- |
| Gender, male/female | 39/12 | 23/20 | 193/64 | 65/12 | 1.000 |
| Age, yrs | 62.5 ± 2.8 | 56.7 ± 1.7 | 58.0 ± 1.7 | 57.6 ± 0.6 | 0.559 |
| Hypertension, n | 35 | 27 | 170 | 45 | 1.000 |
| Hyperlipidemia, n | 33 | 36 | 208 | 58 | 1.000 |
| Diabetes, n | 11 | 8 | 89 | 28 | 1.000 |
| Stroke, n | 5 | 3 | 24 | 13 | 1.000 |
| Smoking, n | 30 | 17 | 157 | 51 | 1.000 |
| Alcohol consumption, n | 20 | 11 | 112 | 31 | 1.000 |
| CHD family history, n | 6 | 10 | 52 | 19 | 1.000 |

Abbreviations: CHD = coronary heart disease, SA = stable angina, UA = unstable angina, AMI = acute myocardial infarction.
